# Tumour-educated platelets for breast cancer detection: biological and technical insights

**DOI:** 10.1038/s41416-023-02174-5

**Published:** 2023-02-10

**Authors:** Marte C. Liefaard, Kat S. Moore, Lennart Mulder, Daan van den Broek, Jelle Wesseling, Gabe S. Sonke, Lodewyk F. A. Wessels, Matti Rookus, Esther H. Lips

**Affiliations:** 1 Division of Molecular Pathology, The Netherlands Cancer Institute, Amsterdam, The Netherlands; 2 Division of Molecular Carcinogenesis, Oncode Institute, The Netherlands Cancer Institute, Amsterdam, The Netherlands; 3 Department of Clinical Chemistry, The Netherlands Cancer Institute, Amsterdam, The Netherlands; 4 Department of Pathology, The Netherlands Cancer Institute, Amsterdam, The Netherlands; 5 Department of Pathology, Leiden University Medical Center, Leiden, the Netherlands; 6 Department of Medical Oncology, Netherlands Cancer Institute, Amsterdam, The Netherlands; 7 Department of EEMCS, Delft University of Technology, Delft, The Netherlands; 8 Department of Psychosocial and Epidemiology Research, The Netherlands Cancer Institute, Amsterdam, The Netherlands

**Keywords:** Breast cancer, Diagnostic markers, Transcriptomics, Tumour biomarkers, Machine learning

## Abstract

**Background:**

Studies have shown that blood platelets contain tumour-specific mRNA profiles tumour-educated platelets (TEPs). Here, we aim to train a TEP-based breast cancer detection classifier.

**Methods:**

Platelet mRNA was sequenced from 266 women with stage I–IV breast cancer and 212 female controls from 6 hospitals. A particle swarm optimised support vector machine (PSO-SVM) and an elastic net-based classifier (EN) were trained on 71% of the study population. Classifier performance was evaluated in the remainder (29%) of the population, followed by validation in an independent set (37 cases and 36 controls). Potential confounding was assessed in post hoc analyses.

**Results:**

Both classifiers reached an area under the curve (AUC) of 0.85 upon internal validation. Reproducibility in the independent validation set was poor with an AUC of 0.55 and 0.54 for the PSO-SVM and EN classifier, respectively. Post hoc analyses indicated that 19% of the variance in gene expression was associated with hospital. Genes related to platelet activity were differentially expressed between hospitals.

**Conclusions:**

We could not validate two TEP-based breast cancer classifiers in an independent validation cohort. The TEP protocol is sensitive to within-protocol variation and revision might be necessary before TEPs can be reconsidered for breast cancer detection.

## Background

The introduction of mammographic screening has enabled detection of breast cancer in asymptomatic women, both in the general population, as well as in women with increased risk of breast cancer who are screened from a young age onwards. Breast cancer screening has increased early detection rates, thereby improving treatment opportunities and decreasing morbidity and mortality [1, 2]. However, mammography is associated with a high number of false positives, leading to unnecessary invasive diagnostic procedures [1, 3, 4]. In addition, sensitivity of mammography is limited in women with high breast density, which may complicate screening of young women with an increased risk of breast cancer [5–7].

Implementation of blood-based markers may not only improve the performance of breast cancer screening but also decrease the costs and increase compliance to the screening programme due to its low burden. Currently, a plethora of blood-based biomarkers is being explored for various applications in breast cancer. Most efforts focus on circulating tumour DNA and circulating tumour cells in the context of treatment response monitoring. However, these biomarkers may be less suited for breast cancer screening, because of their low levels in blood of patients with early stage disease and the heterogeneous nature of breast tumours [8–10].

Previously, differences have been shown between the mRNA content from blood platelets of cancer patients and healthy controls [11, 12]. Aside from their role in haemostasis and wound healing, platelets are also involved in cancer associated processes such as epithelial–mesenchymal transition, metastasis, angiogenesis, and immune evasion. It is hypothesised that the altered platelet mRNA content in cancer patients is the result of specific splicing activity in response to tumour-associated stimuli, in combination with ingestion of tumour-associated mRNA. In the past years, classification algorithms for detection of cancer have been trained based on mRNA profiles from platelets, also referred to as tumour-educated platelets (TEPs) [11, 13–15]. A protocol providing instructions for the complete procedure of TEP collection, processing and particle swarm optimised classifier training was published [16]. Prior to this study, TEP-based classifiers had not been developed for detection of breast cancer. In addition, previous studies did not compare different classifier training methods and did not attempt to reproduce the findings in an independent validation study.

In this multicentre study, the objective was to develop a classification algorithm on TEP mRNA profiles to distinguish patients with breast cancer from healthy controls. In six centres, blood samples were collected and platelets were isolated according to the published protocol [16]. Subsequently, RNA isolation and sequencing was done centrally. Using 71% of the multicentre study population, a classifier was trained according to the published protocol by Best et al. For comparison, an alternative classifier was trained using elastic net regression. Performance of both classifiers was assessed using the remaining 29% of the study population. Lastly, classifier performance was validated in an independent separated case–control study, for which samples were collected exclusively in one of the participating centres.

## Methods

### Experimental model and subject details

#### Human subjects

In total, blood was collected for 553 women of 18 years or older (Supplemental Table 1). Subjects provided written informed consent before blood withdrawal. This study was conducted in accordance with the principles of the Declaration of Helsinki. Approval for this study was obtained from the institutional review board and the ethics committee at each participating hospital (for NKI IRB codes: CFMPB398, CFMPB580, CFMPB617; for other studies, data are publicly available) [13].

#### Method details

For the multicentre set, peripheral whole blood was drawn by venipuncture from breast cancer patients and asymptomatic female controls at the VU University Medical Center, Amsterdam, The Netherlands, the Netherlands Cancer Institute (NKI-AVL), Amsterdam, The Netherlands, the Academic Medical Center, Amsterdam The Netherlands, the Utrecht Medical Center, Utrecht, The Netherlands, the Medical University of Vienna, Vienna, Austria, and Massachusetts General Hospital, Boston, USA. For the single-centre external validation set, peripheral whole blood was drawn by venipuncture from breast cancer patients and asymptomatic female controls at the Netherlands Cancer Institute (NKI-AVL), Amsterdam, The Netherlands. Breast cancer patients were diagnosed by clinical, radiological and pathological examination. Blood of breast cancer patients was collected before start of or during treatment. Asymptomatic controls were self-reported to be free of disease at the moment of blood collection, and were not subjected to additional tests confirming the absence of cancer or other disease. No power calculation was used to determine the number of samples required for algorithm development and validation. Samples for the multicentre set were collected and processed similarly and simultaneously according to a previously published protocol [16]. Samples for the external validation set were collected and processed in the period 2017–2019, following the multicentre study (2015–2017) and applying the same protocol. The researchers were not blinded for case–control status of the multicentre set during processing of the samples, algorithm training and internal validation. For the single-centre external validation set, blinding of platelet pellets was performed by the contributing institute. RNA isolation and sequencing was done blinded and results were linked to case–control status by an independent third party (A. Heemskerk-Gerritsen, Erasmus MC, Rotterdam, The Netherlands). For collection and annotation of clinical data, patient records were manually queried for demographic and clinical variables, i.e. age, sex, type of tumour and stage. All clinical data was anonymised and stored in a secured database.

#### Blood and RNA processing

Whole blood was collected in 6- or 10 mL EDTA-coated Vacutainer tubes and were processed within 12 (part of samples from VU University Medical Center, and the Netherlands Cancer Institute, Academic Medical Center, the Utrecht Medical Center, the Medical University of Vienna) or 48 h (part of samples from VU University Medical Center, and Massachusetts General Hospital, Boston, USA) using standardised protocols as described previously [16]. Platelet rich plasma (PRP) was separated from nucleated blood cells by a 20-min 120 × *g* centrifugation step, after which the platelets were pelleted by a 20-min 360 × *g* centrifugation step. Next, 9/10th of the PRP was removed carefully to reduce the risk of contamination of the platelet preparation with nucleated cells.

Platelet pellets were suspended in RNA (Life Technologies) and after overnight incubation at 4 °C frozen at −80 °C. All RNA isolations and sequencing was performed at the VU University Medical Center, Amsterdam, The Netherlands. For RNA isolation, frozen platelets were thawed on ice and total RNA was isolated using the mirVana miRNA isolation kit (Ambion, Thermo Scientific, AM1560). Platelet RNA was eluted in 30 µL elution buffer. Quality was assessed using the RNA 6000 Picochip (Bioanalyzer 2100, Agilent). Platelet RNA samples with a RIN value >7 and/or distinctive rRNA curves were considered for subsequent analyses.

To obtain sufficient platelet cDNA for robust RNA-seq library preparation, the samples were subjected to cDNA synthesis and amplification using the SMARTer Ultra Low RNA Kit for Illumina Sequencing v3 (Clontech, cat. nr. 634853). Prior to amplification, all samples were diluted to ~500 pg/µL total RNA and again the quality was assessed using the Bioanalyzer Picochip. For samples with a stock yield below 400 pg/µL, a volume of two or more microlitres of total RNA (up to ~500 pg total RNA) was used as input for the SMARTer amplification. Quality control of amplified cDNA was measured using the Bioanalyzer 2100 with DNA High Sensitivity chip (Agilent). All SMARTer cDNA synthesis and amplifications were performed together with a negative control, which was required to be negative by Bioanalyzer analysis. Samples with detectable fragments in the 300–7500 bp region were selected for further processing. All amplified platelet cDNA was first subjected to nucleic acid shearing by sonication (Covaris Inc) and subsequently labelled with single index barcodes for Illumina sequencing using the TruSeq Nano DNA Sample Prep Kit (Illumina, cat nr. FC-121-4001). To account for the low platelet cDNA input concentration, all bead clean-up steps were performed using a 15-min bead-cDNA binding step and a 12-cycle enrichment PCR. All other steps were according to the manufacturer’s protocol. Labelled platelet DNA library quality and quantity were measured using the DNA 7500 chip or DNA High Sensitivity chip (Agilent). High-quality samples with product sizes between 300 and 500 bp were pooled (12–19 samples per pool) in equimolar concentrations for shallow thromboSeq and submitted for 100 bp Single Read sequencing on the Illumina Hiseq 2500 and 4000 platform using version 4 sequencing reagents.

#### Processing of raw RNA-sequencing data

Raw RNA-seq data of platelets encoded in FASTQ-files were subjected to a standardised RNA-seq alignment pipeline, as described previously [16]. In summary, RNA-seq reads were subjected to trimming and clipping of sequence adaptors by Trimmomatic (v. 0.22), mapped to the human reference genome (hg19) using STAR (v. 2.3.0), and summarised using HTSeq (v. 0.6.1), which was guided by the Ensembl gene annotation version 75. Of samples that yielded less than 0.2 × 10^6^ intron-spanning reads in total after sequencing, we again sequenced the original TruSeq preparation of the sample and merged the read counts generated from the two individual FASTQ-files after HTSeq count summarisation. RNAs encoded on the mitochondrial DNA were excluded from downstream analyses. All subsequent statistical and analytical analyses were performed in R (version 4.0.3). Initial QC and filtering operations were performed according to the previously published pipeline (https://github.com/MyronBest/thromboSeq_source_code [16]). Briefly, genes which yielded <30 intron-spanning reads in >90% of the dataset were removed from the count matrix. For each sample, we quantified the number of RNAs for which at least one intron-spanning read was mapped, and excluded samples with <750 detected RNAs. No samples were removed based on this exclusion principle. Next, we performed a leave-one-sample-out cross-correlation analysis, using a correlation threshold of 0.3. Six samples from the control group were excluded due to low inter-sample correlation.

### Quantification and statistical analyses

#### Normalisation strategies

For the elastic net, PSO-SVM, and some downstream analyses, TMM normalisation from the edgeR package [17, 18] was implemented, with some modifications. The calcNormFactors function was adjusted so that both the TMM reference sample and the TMM correction factors could be drawn from a subset of the samples (in this case, the training samples) instead of the entire dataset. This prevented bleed-through of the validation set into the training data. TMM normalisation factors could then be applied during counts-per-million (cpm) and log2 transformation.

In addition to TMM, the PSO-SVM and some downstream analyses utilised RUV factor correction, described in detail in Best et al. [11] and implemented in the publically available repository https://github.com/MyronBest/thromboSeq_source_code. Briefly, the count matrix was normalised using an approach based on the remove unwanted variation (RUV) method, proposed by Risso et al. [19]. The RUVg function employed singular value decomposition to estimate the contribution of covariates of interest (case–control status) and unwanted variation (age, library size, and isolation location). The count matrix was then corrected to compensate for factors of unwanted variation. To prevent removal of unwarranted variation that was correlated with biological signal, two thresholds were applied, based on a Student’s *t* test: (a) the *p* value between RUVg variable and case–control status must exceed 0.01, and (b) the *p* value between RUVg variable and the potentially confounding variable must be less than 0.01.

#### Data partitions for algorithm training

Female breast cancer cases were stratified by stage, followed by random allocation to training, evaluation and internal validation subsets using a 40%–30%–30% schema. For the training and evaluation subsets, a proportionally sized number of age-matched female controls was selected randomly [16]. Age matching between cases and controls was performed using the matchControls function from the R package e1071.

#### Classifier development

The PSO-SVM was trained and evaluated using publicly available code https://github.com/MyronBest/thromboSeq_source_code and is described in detail in Best et al. [11, 16]. Briefly, the PSO-SVM iterates between the training and evaluation data partitions to minimise “1−AUC” as a metric of classifier performance. Algorithm parameters (“particles”) are optimised in an iterative process during which the best performing parameters are communicated within the swarm, allowing the swarm to converge upon the most optimal solution within the search space. The PSO-SVM makes use of this principle to optimise the RUV normalisation thresholds described above, the number of biomarker genes and their rank, and the cost and gamma parameters of the SVM itself. This process relies primarily upon the R packages ppso (for particle swarm implementation), e1071 (for implementation of the SVM), and RUVSeq (for RUV normalisation). For the breast cancer classifier, 100 particles and 10 iterations were employed. Input for the PSO-SVM is RUV-corrected, TMM-normalised, cpm-log2 transformed counts, with age, library size and hospital of origin as RUV factors to correct.

The elastic net was developed using publicly available code and standard methodologies from the caret package in R. The model was produced using the glmnet engine (method = “glmnet”) and ROC as the optimisation metric. Ten-fold cross-validation was employed to optimise the regularisation penalty lambda and the mixing parameter alpha. As the elastic net does not require a separate “evaluation” data partition, “training” and “evaluation” samples were combined to train the elastic net. To reduce the potential impact of sampling artefacts on classifier performance, we additionally trained a second elastic net with leave-one-out cross-validation (LOOCV). This experiment was not possible for the PSO-SVM due to the extremely long runtimes (estimated >1 year) for the PSO-SVM in an LOOCV setting.

Classifier performance on validation sets for both the PSO-SVM and the elastic net was assessed using the R package pROC and ggplot2. Confidence intervals for the ROCs were calculated using the Delong method. Additional performance metrics were extracted via caret::confusionMatrix.

#### Dimensionality analyses

t-SNE visualisations were generated via the R package Rtsne. All t-SNE visualisations were performed on TMM-cpm-log2 corrected counts as described above. Where indicated, some t-SNE plots depicted counts normalised by RUV-correction prior to further normalisation steps.

#### Differential expression

All differential expression tests were performed using the edgeR package in R. TMM normalisation was applied beforehand, using unmodified edgeR methodology. Significance was determined using a quasi-likelihood test. For pairwise comparisons between hospitals, the design formula was ~ age + group + hosp, where “group” indicates case–control status. Additional comparisons were subsetted to produce count matrices containing either cases only, or controls only. These comparisons were made using the design formula ~age + hosp.

#### Batch correction

A comparison of the efficacy of batch correction methods was performed between the ComBat method from the R package sva, and RUV-based correction, described above. With given batch factors (in this case, hospital of origin), RUV was used as a batch correction method in addition to a normalisation method. This was not intended as an exhaustive exploration of all available batch correction methods, but rather as a comparison between RUV-based correction, which was implemented in the previous PSO-SVM-based TEP-cancer publications [11, 16], and a widely used baseline. ComBat was applied as an intercept model on TMM-cpm-log normalised counts.

#### Variance partition

The variance partition analysis was performed using the eponymous R package (variancePartition) [20]. Variance partition applies a linear mixed model to quantify the variance contributed by each element in the design formula, in this case ~Age + (1|hosp) + (1|cancer). As recommended, discrete variables like hospital and case–control status were modelled as random effects when performing this analysis. The overall contribution to variance could subsequently be computed on a per gene basis.

## Results

### Multicentre study: training and assessing performance of the TEP classifiers

**Fig. 1:**
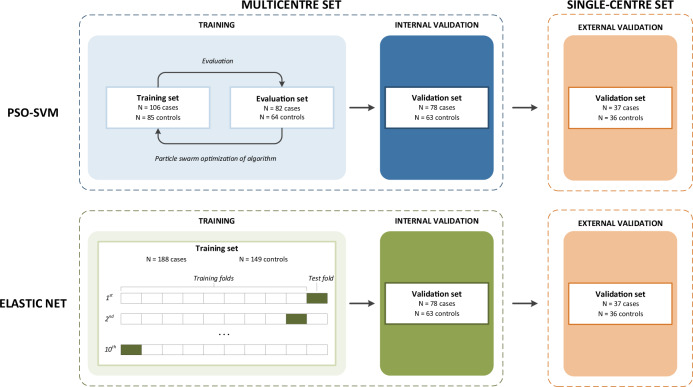
Study design. PSO-SVM particle swarm optimised support vector machine.

We utilised blood platelets from 266 patients with invasive breast cancer and 212 healthy controls from six different centres to develop and assess performance of a breast cancer classifier based on RNA-sequencing data. The study design is visualised in Fig. 1. In line with previously published TEP studies, the multicentre study population was divided into three case–control subsets: a “training”, “evaluation” and “internal validation” subset [16]. After selection of genes with sufficient coverage in the RNA sequencing data, a particle swarm optimised support vector machine (PSO-SVM) classifier was trained on reads that span splice junctions. The PSO-SVM algorithm employs multiple different parameter settings (“particles”) when training the algorithm in the training set and evaluating the performance of each particle in the evaluation set. Because the PSO-SVM learns from the evaluation set when selecting optimal parameters, both training and evaluation samples contributed to training the algorithm. Classifier performance was assessed in the “internal validation” set; these samples did not contribute to the training of the classifier, but were collected, processed and sequenced alongside the samples of the training and evaluation subsets.

Breast cancer cases were stratified by stage, followed by random allocation to the training (*n* = 106, 40.8%), evaluation (*n* = 82, 30.9%) and internal validation (*n* = 78, 29.3%) subsets. For the training and evaluation subsets, a proportionally sized number of age-matched controls was selected randomly, while the remainder of the controls was assigned to the internal validation set. Descriptive statistics for the total multicentre study population and for each of the three subsets are presented in Table 1.

**Table 1:** Descriptive statistics of the training, evaluation, internal validation and external validation sets.

Characteristics	Training	Evaluation	Internal validation	External validation
*Cases, N*	106	82	78	37
Median age (IQR)	51 (40–60)	51 (42–58)	52 (44–61)	60 (53–67)
Stage, *N* (%)				
I	14 (13.2)	11 (13.4)	11 (14.1)	7 (18.9)
II	51 (48.1)	39 (47.6)	38 (48.7)	23 (62.2)
III	14 (13.2)	11 (13.4)	10 (12.8)	5 (13.5)
IV	27 (25.5)	21 (25.6)	19 (24.4)	2 (5.4)
*Controls, N*	85	64	63	36
Median age (IQR)	51 (42–61)	53 (42–60)	52 (24–63)	62 (53–67)

*IQR* interquartile range.

Due to extensive computing time (several weeks per run), it is not feasible to test more than a few randomly assigned data partitions with the PSO-SVM. To assess the impact of sample partitioning on performance, and to provide a baseline performance against which to measure the PSO-SVM, an alternative classifier was trained using an elastic net regression approach (EN). Elastic nets are less computationally intensive and are therefore well suited to nested cross-validation.

For optimal comparison with the PSO-SVM, the EN was trained by 10-fold cross validation using the same data partitions as the PSO-SVM. Since the EN does not require an evaluation set, the training and evaluation sets of the PSO-SVM were combined for training of the EN. Performance of both classifiers in the internal validation set was high, with an area under the curve (AUC) of 0.85 (95% CI 0.79–0.92) for the PSO-SVM and 0.85 (95% CI 0.78–0.91) for the EN classifier (Supplemental Fig. 1).

We additionally tested an EN using a leave-one-out cross-validation (LOOCV) approach, during which a single sample is randomly assigned to be the “test” sample, while the remainder of the dataset is used for training. This process is repeated until every sample has been “left out”, after which performance can be calculated. The LOOCV- EN classifier also performed well, with an AUC of 0.83 (95% CI: 0.79–0.87). Taken together, the PSO-SVM performed well, but not better than the baseline established by the EN classifier (Table 2).

**Table 2:** Performance of EN and PSO-SVM classifiers.

Stage	Model	Features	AUC	95% CI	Sensitivity	Specificity	Precision	Recall	F1
All stages	EN	963	0.85	0.78–0.91	0.79	0.71	0.78	0.79	0.78
Early (I–II)	EN	963	0.81	0.73–0.90	0.73	0.71	0.67	0.73	0.70
Late (III–IV)	EN	963	0.91	0.83–0.98	0.90	0.71	0.59	0.90	0.71
All stages	PSO-SVM	1749	0.85	0.79–0.91	0.86	0.60	0.73	0.86	0.79
Early (I–II)	PSO-SVM	1749	0.81	0.74–0.89	0.80	0.60	0.61	0.80	0.69
Late (III–IV)	PSO-SVM	1749	0.92	0.86–0.98	0.97	0.60	0.53	0.97	0.68

*EN* elastic net, *PSO-SVM* particle swarm optimised support vector machine, *AUC* area under the curve, *95% CI* 95% confidence interval.

### Hospital of origin is a confounder within the multicentre study

**Fig. 2:**
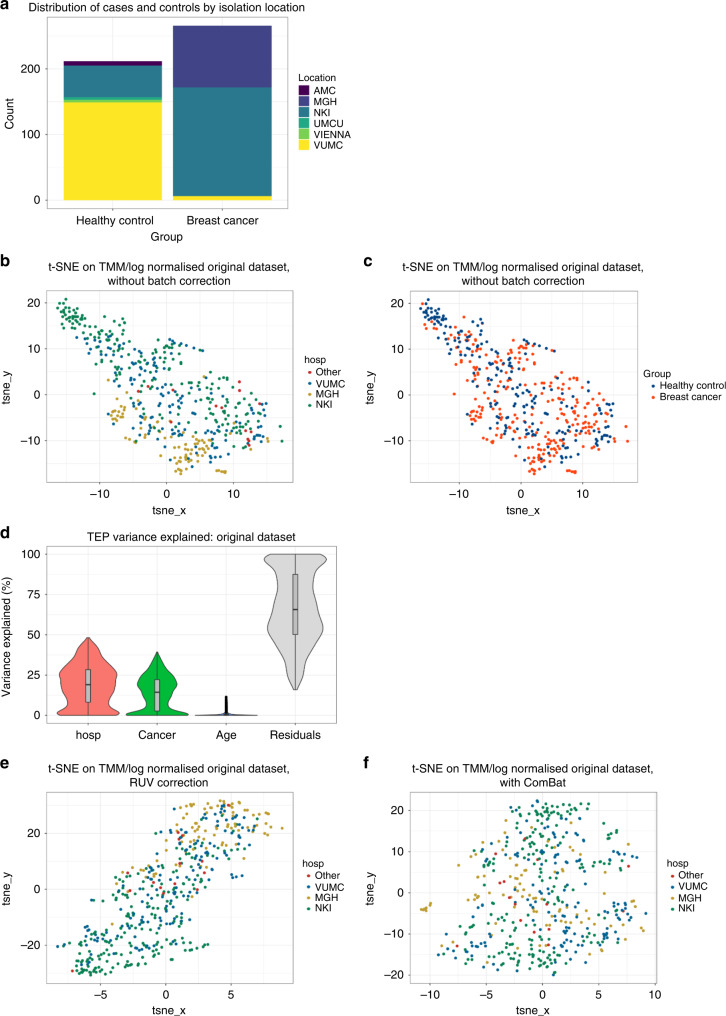
Hospital of origin in a confounder in the multicentre dataset. **a** Numbers of samples of cases and controls contributed by different hospitals. **b** t-SNE plot on the normalised dataset without batch correction labelled for hospital and **c** cancer status. **d** Variance partition violin plot. **e** T-SNE plot on TMM/log normalised dataset labelled for hospital after RUV batch correction and **f** after Combat batch correction.

Although efforts were made to ensure the study design was balanced regarding case–control distribution from participating centres, it was impossible to achieve a completely balanced design due to limited sample availability. Analysis of the multicentre dataset demonstrated a strong correlation between hospital of origin and case–control status (Chi square *p* < 0.001; Fig. 2a). Specifically, controls consisted primarily of samples originating from the VUMC, whereas breast cancer cases were skewed towards NKI and MGH. This observation warranted additional investigation into the potential effect of hospital-related batch effects on platelet RNA profiles.

In dimensionality analyses, it could be seen that there was separation between samples originating from different hospitals (Fig. 2b), but not between cases and controls (Fig. 2c). We quantified the contribution of hospital of origin relative to variance explained by case–control status. This was accomplished using the variancePartition package in R. Briefly, a linear mixed effect model was fit to predict gene expression based on hospital of origin, age and case–control status. As shown in the violin plots in Fig. 2d, an average of 67% of the variance in TEP samples could be attributed to residuals, indicating that the overall fit of the model was poor. Of the remaining variance, 19% was on average explained by hospital of origin, and 14% by case–control status. This indicated that hospital-related batch effects might act as a confounder when training TEP-based classifiers, causing performance to deteriorate when subsequent batches were introduced.

### Platelet activity-related gene expression differs between hospitals

To further investigate the potential confounding influence of hospital-related batch effects, we performed a series of differential expression tests. The KEGG term “platelet activation” was strongly differentially expressed in comparisons between the three centres that contributed the most samples (NKI, VUMC, MGH). Platelet activation was the top upregulated term in MGH vs NKI (FDR = 0.00001, Supplemental Fig. 2a) and VUMC vs NKI (FDR = 7.1e−05, Supplemental Fig. 2b). Comparing VUMC to MGH it was the third most significant term (FDR = 0.03, Supplemental Fig. 2c). The uneven contribution of cases and controls per hospital might also act here as a confounder in differential expression analysis between hospitals. To compensate, we performed expression analysis stratified for case–control status. Given the available numbers, this comparison could be made between VUMC and the NKI for controls, and between MGH and the NKI for cases. Platelet activation was the top enriched term in both comparisons (FDR = 0.001 and FDR = 7.41e−06, respectively, Supplemental Fig. 2d, e), indicating that platelet activation contributed strongly to the batch effect observed between centres.

### Batch correction is insufficient for removing confounding by hospital of origin

In line with previous publications, we initially applied an RUV-based method of batch correction which iteratively corrects the count matrix by removing factors of unwanted variation, such as those correlated with hospital of origin. This method is integrated with the training of the PSO-SVM, and can also be deployed prior to training an EN. However, RUV batch correction is designed to only discard factors of unwanted variation if they are not correlated with case–control status. It is therefore unsuitable for correction of the imbalance in this dataset. Indeed, t-SNE visualisations of the TEP count matrix after iterative RUV correction continued to show substantial clustering based on hospital of origin (Fig. 2e). We therefore compared RUV-based batch correction with ComBat, a well-established batch correction algorithm designed for bulk RNA-seq. ComBat-corrected counts were more diffuse with regards to hospital of origin (Fig. 2f). An EN classifier trained on batch-corrected counts from the multicentre dataset had lower performance when ComBat was applied vs RUV correction (AUCs 0.59 and 0.80 respectively, Supplemental Table 2). Taken together, it would appear that ComBat was able to successfully remove clustering based on hospital of origin, but simultaneously resulted in loss of biological signal. By contrast, RUV correction preserved high classifier performance, but was unable to effectively remove variation due to hospital of origin. Neither method performed adequately for clinical applications, which will involve analysing new batches of samples on an ongoing basis.

### Single-centre classifiers perform poorly on samples from other locations

In order to circumvent hospital-related batch effects, we retrained the classifier on samples originating from a single hospital. Of all the hospitals contributing samples to the multicentre study, only the NKI delivered a sufficient amount of both cases and controls for classifier development (Supplemental Table 1). The EN-classifier was retrained on NKI-only samples using 10-fold cross-validation, balancing case and controls via down-sampling within the cross-validation loop, and predictions made on samples originating from MGH and the VUMC. Centers which contributed less than 10 samples (AMC, UMCU, Vienna) were excluded from this analysis.

**Fig. 3:**
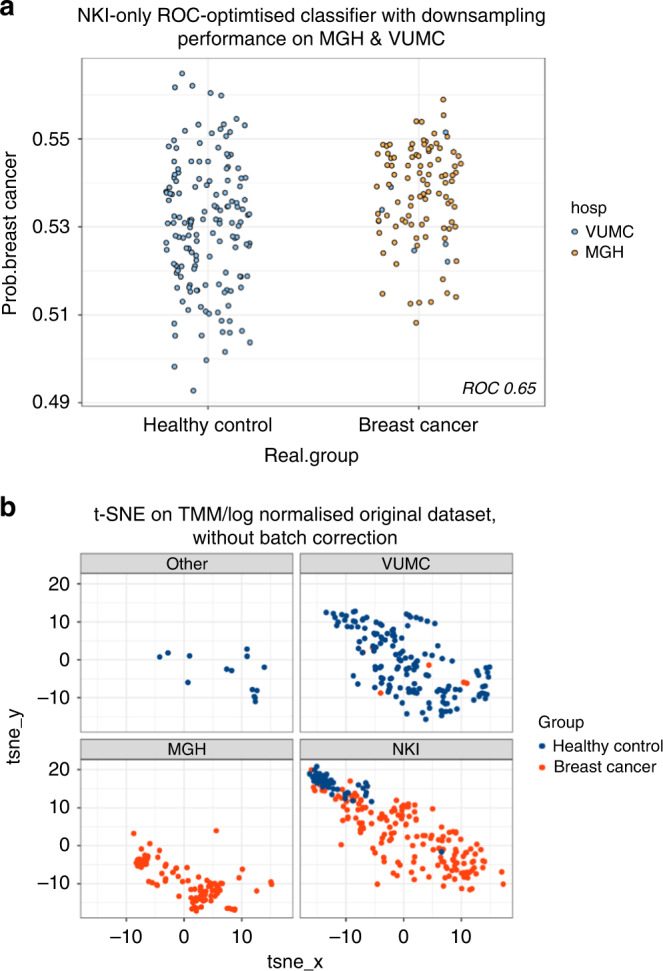
Single-centre classification results for the internal test set samples. **a** Classification results of VUMC and MGH samples when classified by a single centre NKI classifier. **b** t-SNE on TMM/log normalised dataset without batch correction labelled for case–control status for the three hospitals contributing the largest sample numbers.

The single-centre classifier yielded an AUC of 0.65 when predicting on samples that originated from other locations (Supplemental Table 3). Using a 0.5 probability threshold, accuracy was 41%, correctly classifying all 100 breast cancer samples (94 of which originated from MGH) and misclassifying all but 3 of the 149 controls (entirely contributed by the VUMC). Sample classification was predicted with low certainty, with probabilities clustered between 0.49 and 0.58 (Fig. 3a). This was consistent with dimensionality analysis on case–control status when faceted by hospital (Fig. 3b). Within the NKI, cases and controls were well-separated, and the cases from MGH could be superimposed upon the cases from the NKI. However, the controls originating from the VUMC were widely dispersed and largely overlapped with cases of NKI origin. Together, these observations explained why the single-centre classifier demonstrated perfect sensitivity (1.0) but very poor specificity (0.02). Although it is possible to artificially inflate classifier performance by adjusting the probability threshold, doing so would entail mixing training and validation data, and may not be transferable to samples derived from new locations.

### Classifier performance in an external one-centre validation dataset

**Fig. 4:**
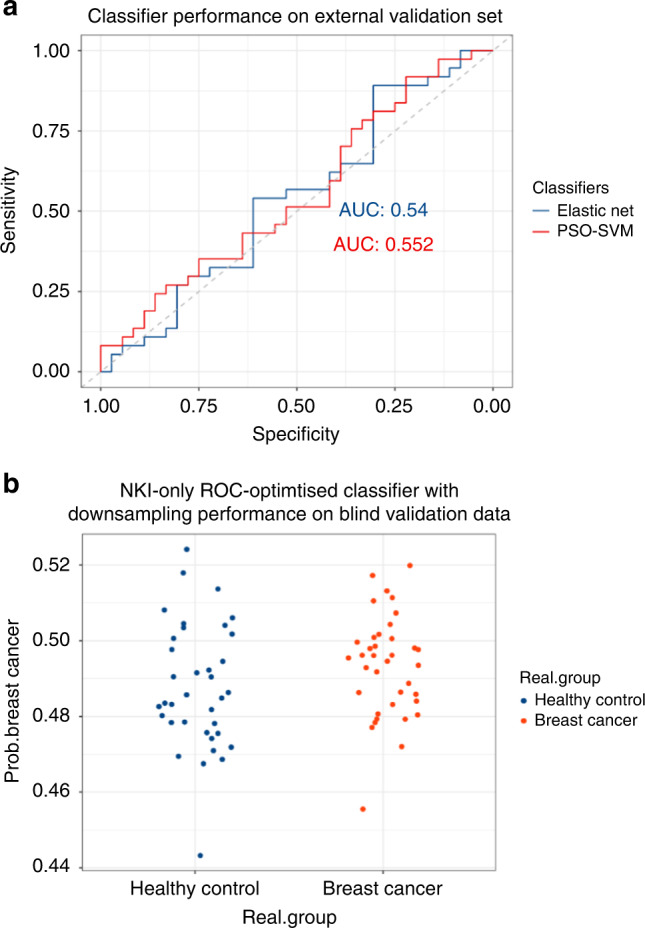
Performance of the classifier in an independent external validation set. **a** ROC curves for the elastic net and PSO-SVM classifiers on the external blinded validation set. **b** Probability distribution of the independent single-centre validation set by EN classification.

Having observed a clear batch effect related to hospital of origin that could not be corrected without loss of classifier performance, and having observed that high performance within a single centre might not validate on samples from other locations, we set out to validate both the PSO-SVM and EN in a new, independent, external validation case–control study (37 cases and 36 age-matched controls) conducted at the NKI after the multicentre study. These samples were collected, processed and sequenced according to the same protocol as applied in the multicentre study. Isolation of mRNA, sequencing and classification was performed blinded, and case–control status was predicted by the PSO-SVM and EN classifiers described in the previous section. Classification labels were subsequently linked to true class labels by an independent third party. Descriptive statistics of the study population are provided in Table 1. Both classifiers showed poor performance, with an AUC of 0.55 (95% CI 0.42–0.69) and 0.54 (95% CI 0.40–0.68) for the PSO-SVM and EN, respectively (Fig. 4a and Supplemental Table 4). Only modest improvement was seen when applying an elastic net classifier trained on batch-corrected counts (RUV is already part of the PSO-SVM). Notably, on the external validation set, ComBat-trained classifiers performed comparable to RUV-trained classifiers (AUCs 0.63 vs 0.62, Supplemental Table 5). This is an indicator that both methods were unable to remove hospital-related batch effects during the training phase, while still preserving biological signal.

Finally, we applied the NKI-only EN classifier to the external validation dataset. Since all samples in the training and validation sets are from the same centre, this analysis should be free of confounding by hospital of origin. Unfortunately, performance on the external validation dataset remained poor, with an AUC of 0.62 (Supplemental Table 6). In this case, probabilities of both cases and controls were distributed around or slightly below the threshold of 0.5 (Fig. 4b). The sensitivity and specificity were 0.27 and 0.72, respectively, with an overall accuracy of 0.49. The poor performance of the NKI-only classifier on a new batch of samples that originated from the same hospital suggests that each new batch effectively represents a new distribution. In line with previous differential expression results, platelet activation was the most statistically significant downregulated KEGG pathway when comparing the external validation samples to the entire multicentre dataset (FDR = 4.5e−05), and also the most significant downregulated pathway when comparing the external validation samples to other samples previously collected from the NKI (FDR = 8.3e–06).

We further investigated the quality of the samples from the independent validation set, to exclude the possibility that the samples were of a lesser quality. We analysed potential erythrocyte and lymphocyte contamination, both by visual inspection analysis of haemoglobin related gene expression and lymphocyte markers (Supplemental Data and Supplemental Figs. 3 and 4). These data showed that there is no reason to expect that erythrocyte or lymphocyte contamination were confounders for the poor classifier performance in the independent validation set.

## Discussion

In the current study, we applied a recently published protocol to develop a particle swarm optimised classifier based on platelet mRNA from blood samples of breast cancer patients and healthy controls. In addition, we compared the performance of the particle swarm-based classifier to an alternative classifier trained on the same samples using elastic net regression. Although initial performance of the classifiers was adequate, post hoc analyses warranted further analyses into hospital of origin as a potential confounder. An attempt to reproduce the findings in an independent, single-centre, external validation set showed low accuracy and was deemed unsuccessful. Our results reveal several issues with the current TEP protocol that need to be addressed in future studies, which are summarised in Table 3.

**Table 3:** TEP-related issues highlighted in our study and potential solutions for future studies.

Issue to resolve	Potential solutions
Optimisation of study design	• Balanced study design with matched cases and controls from the same hospital
	• Extensive collection of detailed information on clinical variables such as treatment status
Prevention of batch effects related to sample processing	• Detailed registration of sample processing, including processing time and blood tube volume
	• Dedicated trained technicians for sample processing
	• Homogenisation of blood processing, including blood tube volume and processing time
	• Centralised processing whenever feasible
	• Addition of leukodepletion step in the protocol
	• Standardised evaluation of lymphocyte and erythrocyte contamination
Ensuring detection of tumour-specific signal if present	• Increase amounts of RNA subjected to PCR
	• Training on all reads, not just spliced reads

First, we found that gene expression in platelets was heavily influenced by hospital of origin, despite all hospitals using the same platelet-processing protocol, and despite technicians at the NKI-AVL being trained in platelet isolation by technicians of the VUMC. Specifically, platelet activation related genes were differentially expressed between the three largest contributing hospitals, independent of case–control status. This may possibly be explained by differences in processing allowed for within the protocol, such as the time allowed between blood withdrawal and platelet isolation. According to the protocol, whole blood can be stored at room temperature for up to 48 h before platelet isolation [16]. However, previous studies have shown that platelet activity is increased with longer time to processing and higher temperature [21]. A previous TEP study in non-small cell lung cancer showed stable levels of platelet activation markers such as P-selectin and CD63 for different processing times, but the sample size was small (*n* = 6) and the same study did show enhanced platelet activation related gene expression in controls vs cases, which was not discussed in depth [11]. Since data on exact duration of processing was not registered for samples from some centres, we could not formally analyse this variable in our study. However, in practice we observed that in the NKI, samples are processed faster than in the VUMC (2–6 h vs 12–24 h). Therefore, further research to investigate the magnitude of processing time as a confounding factor is warranted. Moreover, other within-protocol differences may also play a role. According to the protocol, blood tube volumes of 4, 6 or 10 mL are accepted. In the NKI, 10 mL blood tubes are used, whereas in the VUMC, 6 mL blood tubes are used. In a previous study comparing several protocols for isolation of platelet-rich-plasma (PRP) for treatment purposes, it was shown that the platelet concentration correlates with blood tube volume and centrifugation speed [22]. Although the aforementioned study did not investigate the current TEP protocol, and no platelet RNA sequencing was performed, it is possible that TEP gene expression may be also be influenced by these factors.

To bypass the confounding effect of hospital we trained and tested a classifier solely on samples from a single hospital, which did not result in improved classifier performance. This suggests that batch effects are not only relevant between hospitals, but also within hospitals, indicating that the protocol is highly sensitive to subtle variations in execution other than the variables mentioned earlier. Moreover, batch correction as implemented in the classifier training portion of the protocol, proved unsuccessful in removing hospital-related confounding. In future studies these batch-related issues may in part be addressed by centralised sample processing. However, sample transportation time and temperature may influence TEP gene expression results, which is another concern. In addition, before TEP-based biomarkers can be implemented clinically, showing analytical validity of the protocol by reproducing study results in other centres is mandatory.

Given the poor performance of the classifiers in the external validation set, and the lack of a leukodepletion step in the TEP protocol underlying our study, we investigated sample quality in post-hoc analyses. While the samples in the external validation set showed higher CD3 subunit expression than the samples in the original set, indicative of leucocyte contamination, no correlation was found with incorrect classification of the samples. Previous multi-centre studies based on the current TEP protocol have resulted in highly accurate classifiers without a leukodepletion step, but expression of leucocyte-specific markers was not reported [11, 13–15]. Given the absence of a predictive relationship between putative leucocyte contamination and sample misclassification, we consider it unlikely that leukodepletion alone will be sufficient to rescue TEP classifier performance. Nevertheless, we do recommend evaluation of the extent and influence of contamination in future TEP studies.

The majority of the gene expression signal in our study was attributable to residual factors, which might be explained by variables such as use of medication or presence of other systemic disease. Our study contains some individuals with breast cancer who had started treatment at the time of blood withdrawal. While previous studies have shown that the TEP profile seems to normalise after tumour resection, impact of systemic treatment on platelet gene expression has not been investigated [15]. Given that most chemotherapeutic drugs suppress the bone marrow with thrombocytopenia as a common side effect, an effect on the platelet gene expression profile seems probable. In our study, we were unable to analyse the effect of treatment on the platelet profile, since data was not available for most patients. However, given that our study was set up to include patients at the time of diagnosis, the number of patients that are pre-treated is likely to be low, and we expect the majority to still have detectable disease upon blood withdrawal. In addition, previous studies have been able to successfully train classifiers with inclusion of patients who had started treatment [11, 13]. Besides treatment effects, it has been shown that the platelet transcriptome is altered in patients with severe acute disease such as sepsis and myocardial infarction, but also chronic disease such as lupus and chronic kidney disease [23–26]. Changes in the platelet mRNA profile have also been linked to obesity, race, and medication use [27–29]. In our study, co-morbidities were not reported for breast cancer patients. Healthy controls were self-reported to be free of disease, and they were not asked about medication use or subjected to any diagnostic investigations to confirm absence of disease. Therefore, we cannot exclude the presence of (latent) disease and response bias. A previous study evaluating TEP RNA in healthy subjects showed minor differences based on age and sex, storage time and library size [30], and showed clustering based on immune-related genes potentially related to infection. Although it was deemed unlikely by the authors that the small variability resulting from these factors would have a biological effect, this may significantly complicate development of classifiers intended for large-scale screening. Ideally, future studies should extensively document the health state and treatment status of participating subjects and the relation to TEP gene expression.

In our study, the top genes that were selected in the PSO-SVM and EN classifiers did not correspond to the top genes that were associated with case–control status, indicating that there is no substantial signal in the data that can be utilised for reliable classifier training. In addition, the top classifier features do not represent known breast cancer biology, which raises uncertainty on their utility as biomarkers. The lack of a breast cancer signal may in part be explained by the small amounts of TEP mRNA subjected to PCR, which may give rise to PCR artefacts. In addition, only reads that span the splice junction were used for feature selection, which left out the majority of mRNA sequencing information that might be useful for classification.

Our findings may have impact on the interpretation of previously published papers on TEP RNA based classifiers. A pan-cancer detection algorithm that was published in 2015 showed a detection accuracy of 96% using an internal validation subset, and correct tumour classification over six tumour types of 71% [13]. Similarly, also using internal validation subsets a PSO-SVM trained for detection of non-small-cell lung cancer showed 88 and 81% classification accuracy in late stage and early stage cancer, respectively [11]. However, in the subset used for training of the non-small cell lung cancer classifier, hospital of origin was also unevenly distributed between cases and controls. Therefore, the classifier might be subject to a similar hospital-of-origin batch effect, although the imbalance was less pronounced than in our dataset. For our study, we followed the protocol as published by Best et al. in Nature Protocols, which is the same protocol used for aforementioned studies [16]. In contrast to the previous studies, our population included mainly early stage patients. Previous studies investigating other blood based biomarkers in breast cancer have shown that it is difficult to reliably reproduce biomarkers in early stage breast cancer. Classification accuracy may be better when more late stage patients are included in the training set. However, since the aim of our study was to find a biomarker suited for screening, a potential classifying algorithm should be able to sufficiently recognise early stage breast cancer.

In conclusion, we were unable to successfully validate a TEP RNA based breast cancer detection classifier in a single-centre, independent, blinded study. Both elastic net and particle swarm-based classifiers performed poorly. The gene expression profile was severely influenced by hospital of origin and other factors unrelated to case–control status, suggesting that the wet lab protocol is highly sensitive to within-protocol variations in execution. Therefore, thorough revision of the protocol is necessary before TEP RNA based classifiers can be reconsidered for breast cancer detection in the future.

## Supplementary information

Data Supplement

Supplemental Table 1

Supplemental Tables 2-6

Supplemental Figures

## Data Availability

The datasets generated during and/or analysed during the current study are deposited at the European Genome-phenome Archive (EGA) under the accession numbers EGAS00001006821 and EGAD00001009790.
