# Correction To: Tumour-educated platelets for breast cancer detection: biological and technical insights

**DOI:** 10.1038/s41416-023-02371-2

**Published:** 2023-07-24

**Authors:** Marte C. Liefaard, Kat S. Moore, Lennart Mulder, Daan van den Broek, Jelle Wesseling, Gabe S. Sonke, Lodewyk F. A. Wessels, Matti Rookus, Esther H. Lips

**Affiliations:** 1 Division of Molecular Pathology, The Netherlands Cancer Institute, Amsterdam, The Netherlands; 2 Division of Molecular Carcinogenesis, Oncode Institute, The Netherlands Cancer Institute, Amsterdam, The Netherlands; 3 Department of Clinical Chemistry, The Netherlands Cancer Institute, Amsterdam, The Netherlands; 4 Department of Pathology, The Netherlands Cancer Institute, Amsterdam, The Netherlands; 5 Department of Pathology, Leiden University Medical Center, Leiden, the Netherlands; 6 Department of Medical Oncology, Netherlands Cancer Institute, Amsterdam, The Netherlands; 7 Department of EEMCS, Delft University of Technology, Delft, The Netherlands; 8 Department of Psychosocial and Epidemiology Research, The Netherlands Cancer Institute, Amsterdam, The Netherlands

**Keywords:** Breast cancer, Diagnostic markers, Transcriptomics, Tumour biomarkers, Machine learning

Correction to: *British Journal of Cancer* 10.1038/s41416-023-02174-5, published online 10 February 2023

In this article the data availability statement was incorrectly given. “EGAS0000100682” should have been “EGAS00001006821”.

The correct statement as follows.

The datasets generated during and/or analysed during the current study are deposited at the European Genome-phenome Archive (EGA) under the accession numbers EGAS00001006821 and EGAD00001009790.

The original article has been corrected.

