# Supplementary material for: Tumour-educated platelets for breast cancer detection: biological and technical insights
Source: Br J Cancer. 2023 Feb 10;128(8):1572–81. doi: 10.1038/s41416-023-02174-5 (PMC10070267; doi:10.1038/s41416-023-02174-5)
Supplement: Supplementary file 1 — Data Supplement [file 41416_2023_2174_MOESM1_ESM.docx]

**Data Supplement**

**Assessment of quality of the validation samples: erythrocyte and lymphocyte contamination**

The observation of pronounced batch effects in samples originating from the same hospital and processed according to the same protocol warranted further investigation into the quality of the samples. To exclude the possibility that the samples of the external validation set were of a lesser quality than the sample of the multicenter study, we analysed potential erythrocyte and lymphocyte contamination. Since only one of the two available platelet pellets was used for sequencing, the remaining pellet was retrospectively subjected to visual scoring and classified as red (high erythrocyte contamination suspected, n = 13), pink (light erythrocyte contamination suspected, n = 27) or white (no erythrocyte contamination suspected, n = 33). Erythrocyte contamination was studied further by evaluating hemoglobin related gene expression. Expression of hemoglobin subunit beta (HBB) was slightly higher in red-colored samples, but hemoglobin subunit gamma-2 (HBG2) was not (**Sup Fig 3a & b**). No relation was observed between redness of the samples and case-control status (Pearson’s Chi-Squared Test, p = 0.72) or correct classification of the samples for both the PSO-SVM and the EN classifier (Pearson’s Chi-Squared Test, p = 0.63 and p = 0.33, respectively). In addition, there was no association between HBB or HBG2 expression and misclassification (**Sup Fig 3c & d**).

No material was available for assessing pellet discoloration in samples used for training the classifiers for comparison with the blind validation samples. However, a comparison can be made based on RNA expression of HBB and HBG2 in samples originating from different isolation locations (**Sup Fig 3e & f**). Although HBB expression was higher on average in external validation samples than in the multicenter study, the HBB expression of the majority of the validation samples fall within the second and third quantiles of the multicenter study samples. By contrast, HBG2 expression was actually lower in the external validation samples than in the samples of the multicenter study.

Taken together, there is no reason to assume that pellet discoloration is indicative of meaningful erythrocyte contamination, or that hemoglobin expression is a confounder for classifier performance. We nevertheless investigated whether negative classifier performance could be attributed to discolored samples. After excluding the red colored samples, the AUC of receiver operating characteristic (ROC) curve was 0.55 (95% CI 0.40-0.70) for the PSO SVM and 0.57 (0.42-0.72) for the EN classifier in the external validation set (**Sup Fig 3g**), representing no significant improvement in performance.

Similarly, we investigated potential lymphocyte contamination by assessing CD3 subunit expression as a marker for lymphocytes. CD3 subunit expression was on average higher in the external validation set than in the multicenter study **(Sup Figs 4a & b).** CD3 subunits were not ranked highly among the features selected by either the PSO-SVM or the elastic net, nor are lymphocyte related terms ranked highly in differential expression tests between different institutions. In addition, there was no association between CD3 expression and misclassification (**Sup Fig 4c & d**). Therefore, it is unlikely that lymphocyte contamination is responsible for the poor classifier performance in the external validation set. Importantly, platelets can absorb RNA from their environment. Therefore, the presence of hemoglobin and CD3 transcripts may not represent contamination, but rather normal TEP biology.
