# Supplementary material for: Tumour-educated platelets for breast cancer detection: biological and technical insights
Source: Br J Cancer. 2023 Feb 10;128(8):1572–81. doi: 10.1038/s41416-023-02174-5 (PMC10070267; doi:10.1038/s41416-023-02174-5)
Supplement: Supplementary file 3 — Supplemental Tables 2-6 [file 41416_2023_2174_MOESM3_ESM.docx]

**Sup Tbl 2. Detailed elastic net performance on internal validation set following batch correction**

| method | dataset | AUC | 95% CI | Accuracy | Sensitivity | Specificity | PPV | NPV | F1 | Kappa |
| --- | --- | --- | --- | --- | --- | --- | --- | --- | --- | --- |
| ComBat | internal validation | 0.5936 | 0.49917-0.68805 | 0.5816 | 0.7949 | 0.3175 | 0.5905 | 0.5556 | 0.6776 | 0.1172 |
| RUV | internal validation | 0.804 | 0.73159-0.87647 | 0.7447 | 0.8205 | 0.6508 | 0.7442 | 0.7455 | 0.7805 | 0.4771 |

*Abbreviations: AUC = Area Under the Curve; 95% CI = 95% Confidence Interval; PPV = Positive Predictive Value; NPV = Negative Predictive Value*

**Sup Tbl 3. Detailed results of single-center classifier**

| train_hosp | predict_hosp | AUC | 95% CI | Accuracy | Sensitivity | Specificity | PPV | NPV | F1 | Kappa |
| --- | --- | --- | --- | --- | --- | --- | --- | --- | --- | --- |
| NKI | VUMC & MGH | 0.6552 | 0.58699-0.72334 | 0.4137 | 1 | 0.0201 | 0.4065 | 1 | 0.578 | 0.0162 |

*Abbreviations: AUC = Area Under the Curve; 95% CI = 95% Confidence Interval; PPV = Positive Predictive Value; NPV = Negative Predictive Value*

**Sup Tbl 4. Classifier performance on external validation set**

| Model | Validation | AUC | 95% CI | Sensitivity | Specificity | PPV | NPV |
| --- | --- | --- | --- | --- | --- | --- | --- |
| elastic net | blindNKI | 0.5398 | 0.40461-0.67497 | 0.4324 | 0.6111 | 0.5333 | 0.5116 |
| pso-svm | blindNKI | 0.5518 | 0.41782-0.68578 | 0.7568 | 0.3333 | 0.5385 | 0.5714 |

*Abbreviations: PSO-SVM = Particle Swarm Optimized Support Vector Machine; AUC = Area Under the Curve; 95% CI Confidence Interval; PPV = Positive Predictive Value; NPV = Negative Predictive Value*

**Sup Tbl 5. Detailed performance of elastic net on external validation following batch correction**

| method | validation | validation_data | AUC | 95% CI | Accuracy | Sensitivity | Specificity | PPV | NPV | F1 | Kappa |
| --- | --- | --- | --- | --- | --- | --- | --- | --- | --- | --- | --- |
| ComBat | blind validation | ComBat ROC on Blind | 0.6344 | 0.50459-0.76418 | 0.5616 | 0.7838 | 0.3333 | 0.5472 | 0.6 | 0.6444 | 0.1178 |
| RUV | blind validation | RUV ROC on blindval | 0.6179 | 0.48764-0.7481 | 0.5753 | 0.2703 | 0.8889 | 0.7143 | 0.5424 | 0.3922 | 0.1578 |

*Abbreviations: AUC = Area Under the Curve; 95% CI = 95% Confidence Interval; PPV = Positive Predictive Value; NPV = Negative Predictive Value*

**Sup Tbl 6. Detailed performance of the NKI-only elastic net classifier on the external validation set**

| train_hosp | predict_hosp | AUC | 95% CI | Accuracy | Sensitivity | Specificity | PPV | NPV | F1 | Kappa |
| --- | --- | --- | --- | --- | --- | --- | --- | --- | --- | --- |
| NKI | blindNKI | 0.6164 | 0.4835-0.74924 | 0.4932 | 0.2703 | 0.7222 | 0.5 | 0.4906 | 0.3509 | -0.0075 |

*Abbreviations: AUC = Area Under the Curve; 95% CI = 95% Confidence Interval; PPV = Positive Predictive Value; NPV = Negative Predictive Value*
