# Supplementary material for: Tumour-educated platelets for breast cancer detection: biological and technical insights
Source: Br J Cancer. 2023 Feb 10;128(8):1572–81. doi: 10.1038/s41416-023-02174-5 (PMC10070267; doi:10.1038/s41416-023-02174-5)

Supplemental Figure 1. Receiver operating curves for the internal validation set showing performance of both the particle swarm optimized support vector machine and the elastic net-based classifier

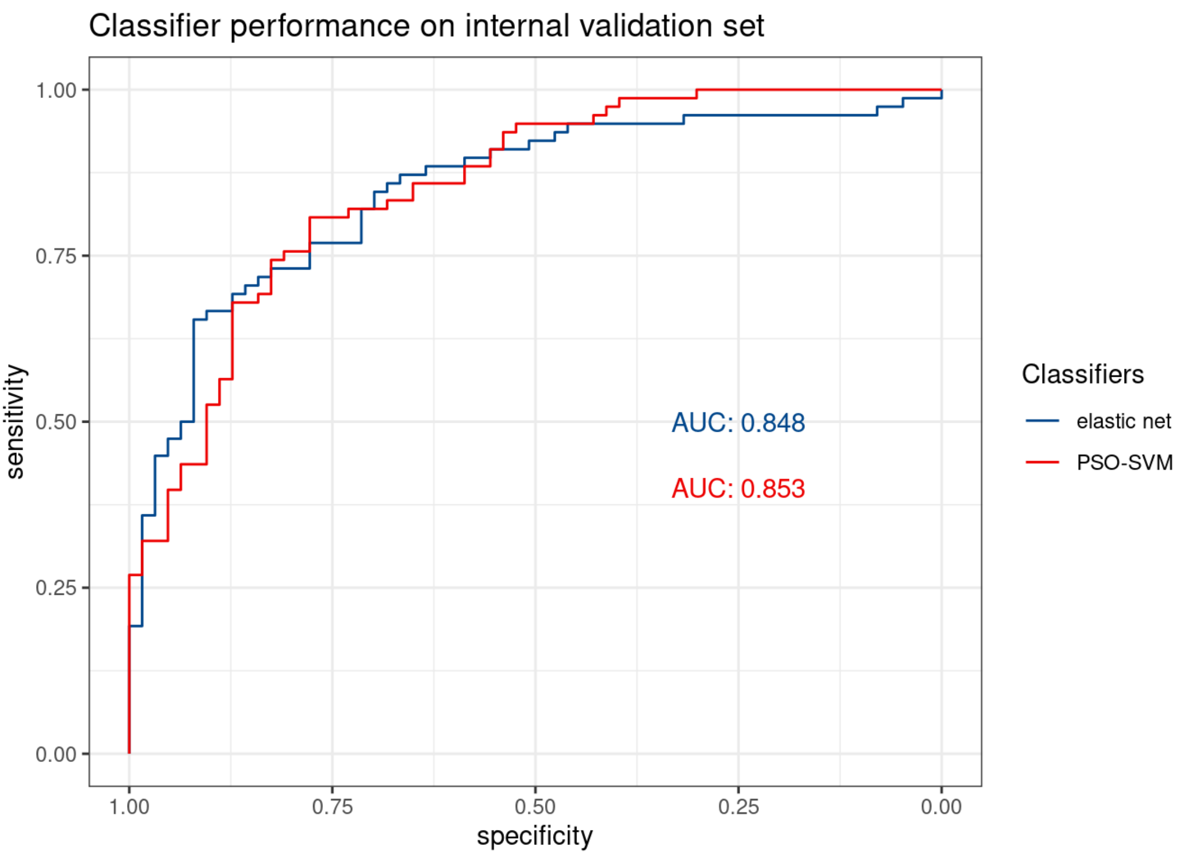

Supplemental Figure 2. Platelet activity between hospitals

a

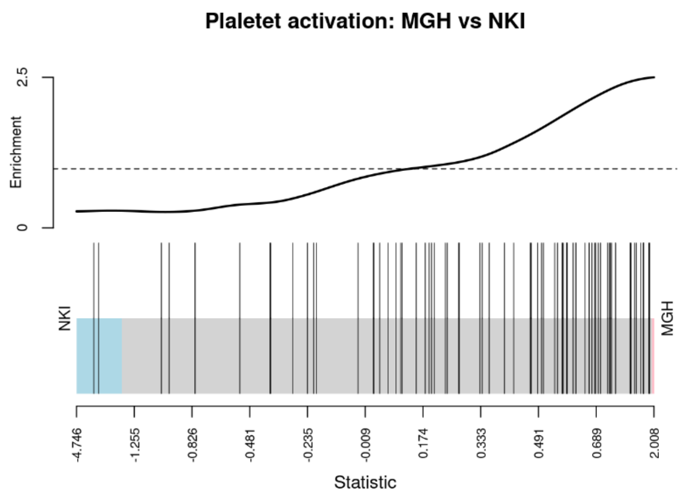

b

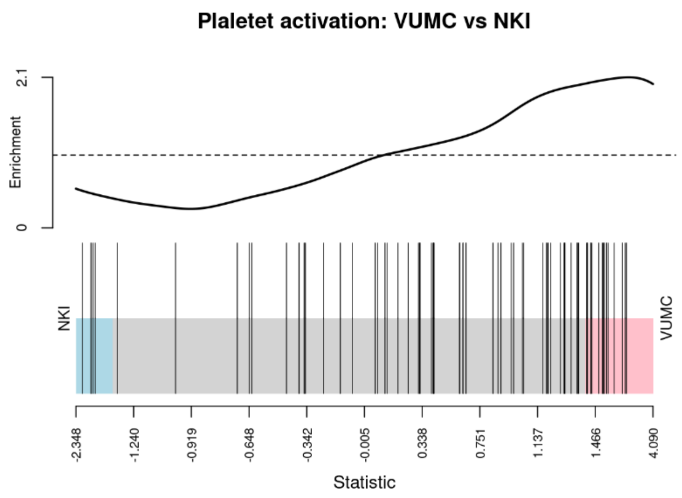

c

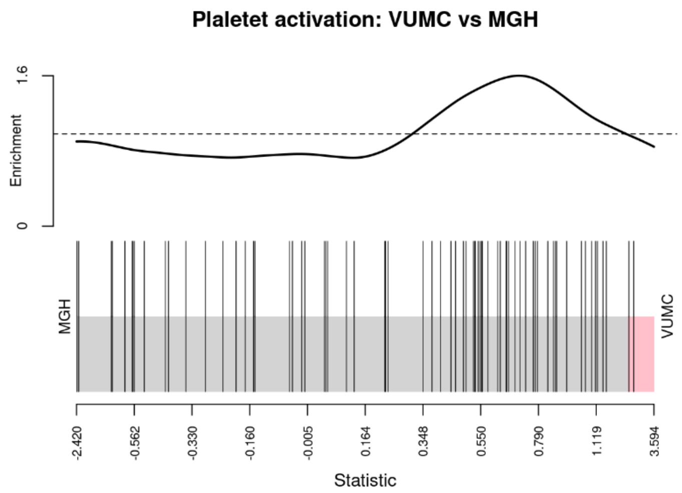

d

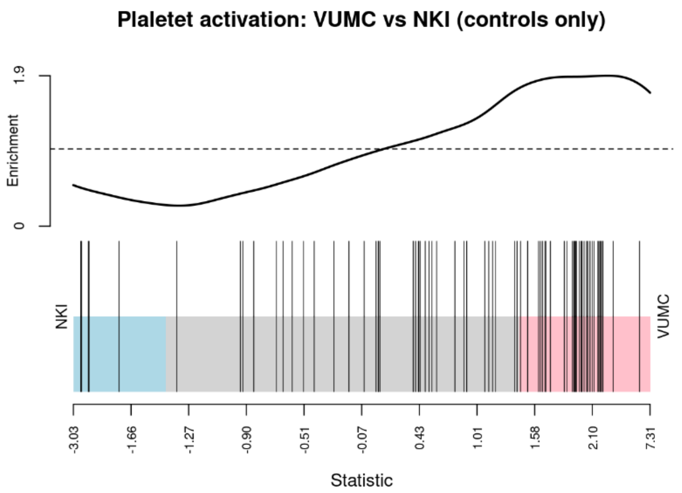

e

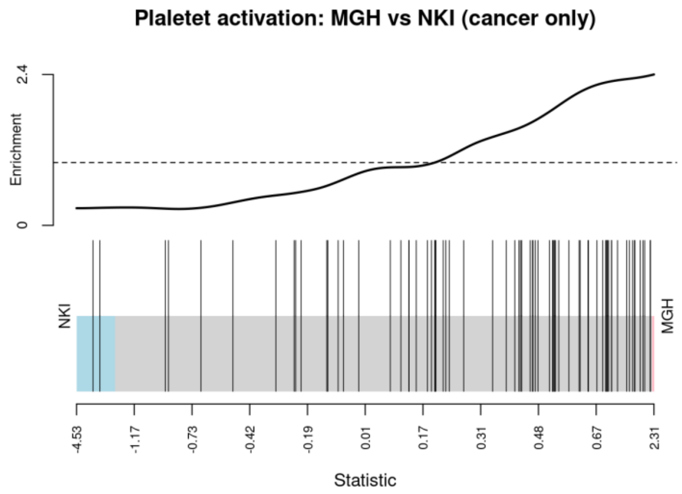

Supplemental Figure 3. Quality control of blinded validation cohort for erythrocyte contamination *(a) HBB and TEP pellets by visual discoloration. (b) HBG2 and TEP pellets by visual discoloration. (c) HBB expression by classification status in the external validation set (left panel) and multicenter dataset (right panel). Shown is the p value from a Wilcoxon rank sum test. (d) HBG2 expression by classification status in the external validation set (left panel) and multicenter dataset (right panel). Shown is the p value from a Wilcoxon rank sum test. (e) HBB expression per hospital. (f) HBG2 expression per hospital. (g) Elastic net classifier performance in the external validation set when red-tinted samples were excluded.*

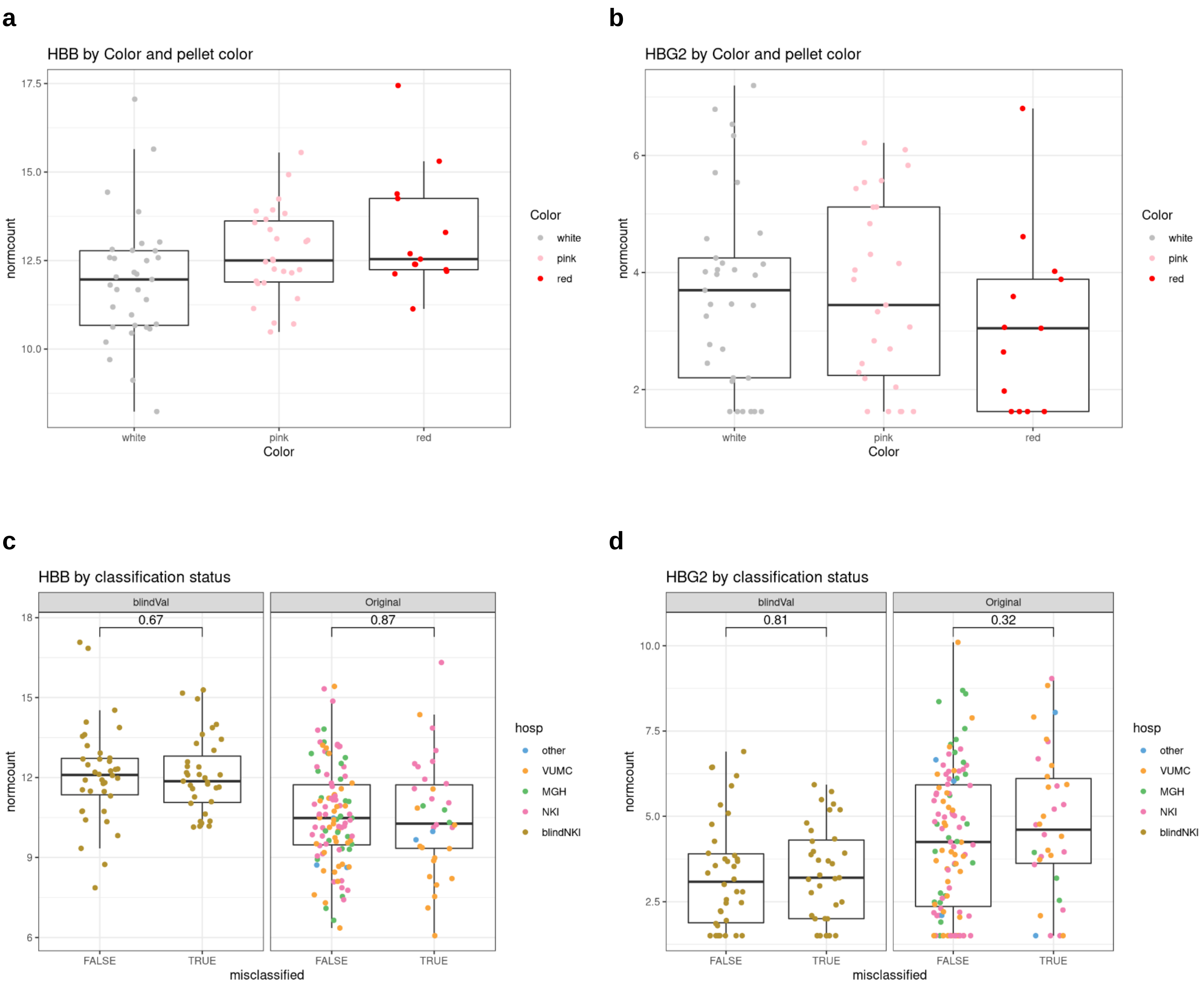

e

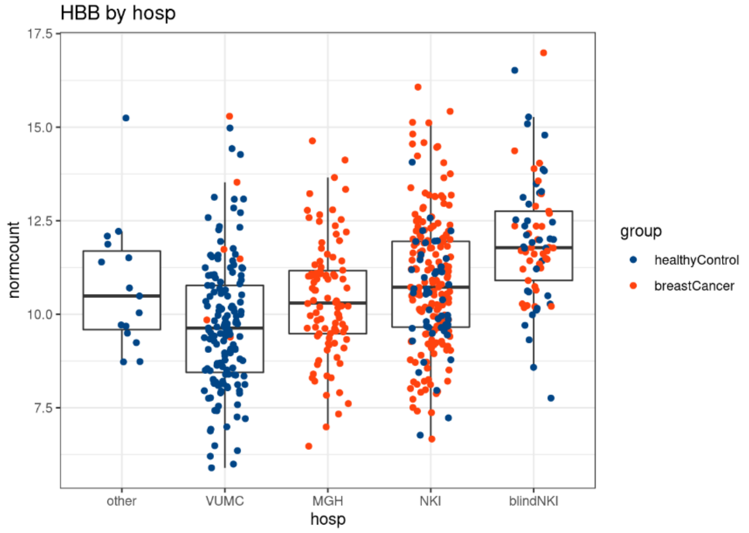

HBB by hosp
17.5
15.0
12.5
10.0
7.5
normcount
other
VUMC
MGH
NKI
blindNKI
hosp
group
healthyControl
breastCancer

f

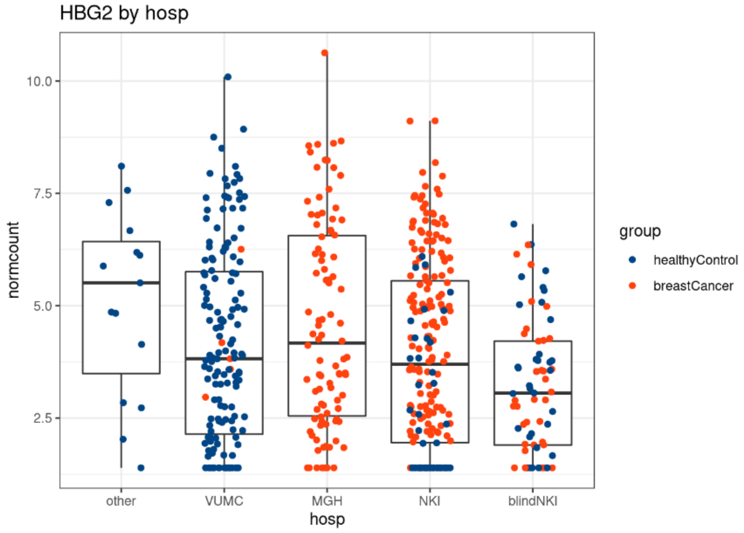

HBG2 by hosp
10.0
7.5
5.0
2.5
normcount
other
VUMC
MGH
NKI
blindNKI
hosp
group
healthyControl
breastCancer

g

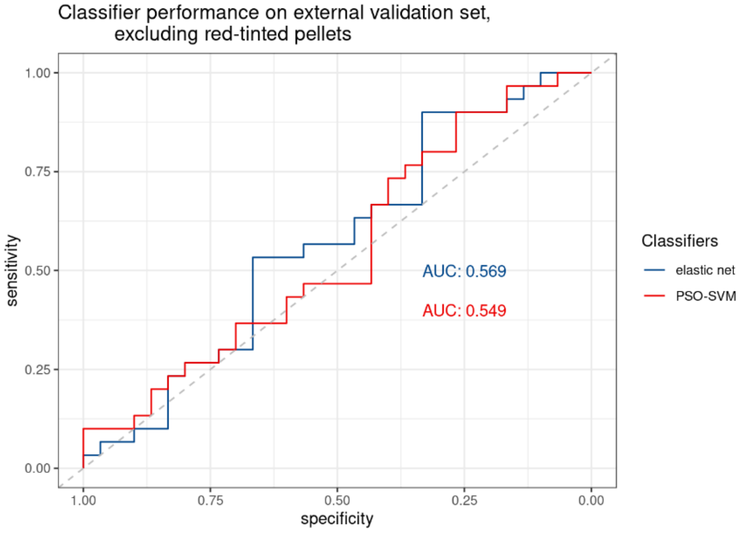

Classifier performance on external validation set, excluding red-tinted pellets
1.00
0.75
0.50
0.25
0.00
sensitivity
1.00
0.75
0.50
0.25
0.00
specificity
AUC: 0.569
AUC: 0.549
Classifiers
elastic net
PSO-SVM

Supplemental Figure 4. Quality control of blinded validation cohort for lymphocyte contamination *(a) CD3D expression by hospital of origin. (b) CD3E expression by hospital of origin. (c) CD3D expression by classification status in the external validation set (left panel) and multicenter dataset (right panel). Shown is the p value from a Wilcoxon rank sum test. (d) CD3E expression by classification status in the external validation set (left panel) and multicenter dataset (right panel). Shown is the p value from a Wilcoxon rank sum test.*

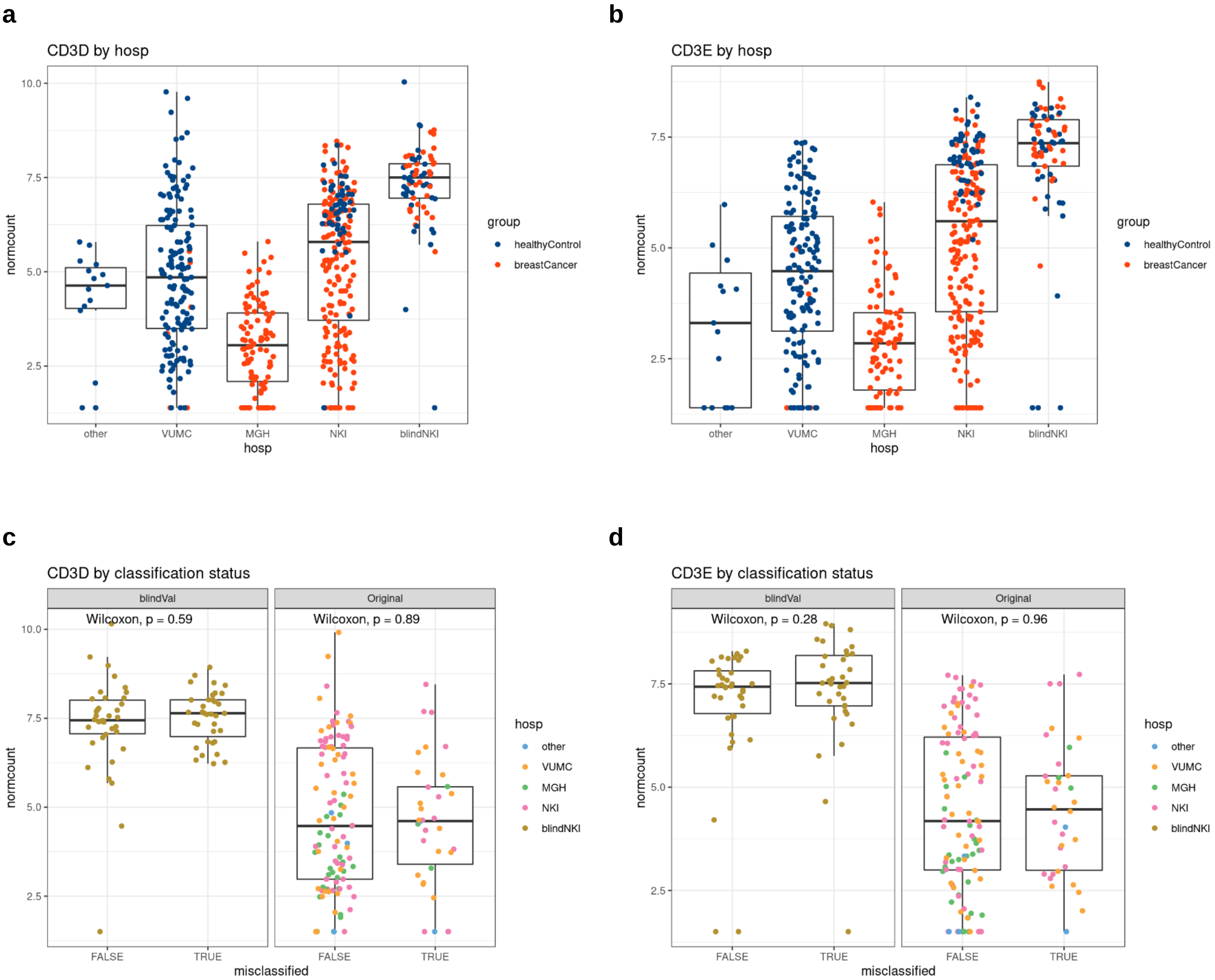

Supplement: Supplementary file 4 — Supplemental Figures [file 41416_2023_2174_MOESM4_ESM.pdf]
